# Effects of Aerobic Exercise Therapy through Nordic Walking Program in Lactate Concentrations, Fatigue and Quality-of-Life in Patients with Long-COVID Syndrome: A Non-Randomized Parallel Controlled Trial

**DOI:** 10.3390/jcm13041035

**Published:** 2024-02-11

**Authors:** Sofía Laguarta-Val, David Varillas-Delgado, Ángel Lizcano-Álvarez, Alberto Molero-Sánchez, Alberto Melian-Ortiz, Roberto Cano-de-la-Cuerda, Carmen Jiménez-Antona

**Affiliations:** 1 Department of Physical Therapy, Occupational Therapy, Rehabilitation and Physical Medicine, Faculty of Health Sciences, Universidad Rey Juan Carlos, Alcorcon, 28922 Madrid, Spain; sofia.laguarta@urjc.es (S.L.-V.); alberto.molero@urjc.es (A.M.-S.); roberto.cano@urjc.es (R.C.-d.-l.-C.); carmen.jimenez@urjc.es (C.J.-A.); 2 Department of Exercise and Sport Science, Faculty of Health Sciences, Universidad Francisco de Vitoria, 28223 Pozuelo, Spain; 3 Department of Nursing and Stomatology, Faculty of Health Sciences, Universidad Rey Juan Carlos, Alcorcon, 28922 Madrid, Spain; angel.lizcano@urjc.es; 4 Faculty of Nursing and Physiotherapy, Universidad Pontificia de Salamanca, 28015 Madrid, Spain; amelianor@upsa.es

**Keywords:** Long-COVID, COVID-19, fatigue, quality of life, lactate concentration, aerobic exercise, Nordic Walking program

## Abstract

Background: Long-COVID syndrome comprises a variety of signs and symptoms that develop during or after infection with COVID-19 which may affect the physical capabilities. However, there is a lack of studies investigating the effects of Long-COVID syndrome in sport capabilities after suffering from COVID-19 infection. The purpose of the study was to evaluate and compare lactate concentration and quality of life (QoL) in patients with Long-COVID with those who have not developed non-Long-COVID during Nordic walking exercise therapy. Methods: Twenty-nine patients (25.5 ± 7.1 years) took part in a non-randomized controlled trial, divided into two groups: a Long-COVID group (*n* = 16) and a non-Long-COVID control (*n* = 13). Patients were confirmed as having Long-COVID syndrome if they experienced fatigue or tiredness when performing daily activities and worsening of symptoms after vigorous physical or mental activity. All participants underwent a 12-week Nordic Walking program. Lactate concentration after exercise and distance covered during all sessions were measured. Pre- and Long-Nordic Walking program, the Modified Fatigue Impact Scale (MFIS), the Short Form 36 Health Survey (SF-36), and EURO QoL-5D (EQ-ED) were administered to assess fatigue and quality of life, respectively. Results: There was a lactate concentration effect between groups (F = 5.604; *p* = 0.024). However, there was no significant effect as a result of the session (F = 3.521; *p* = 0.121) with no interaction of group × session (F = 1.345; *p* = 0.414). The group main effect (F = 23.088; *p* < 0.001), time effect (F = 6.625; *p* = 0.026), and group × time (F = 4.632; *p* = 0.002) interaction on the SF-36 scale were noted. Also, there were a significant group main effect (F = 38.372; *p* < 0.001), time effect (F = 12.424; *p* = 0.005), and group × time interaction (F = 4.340; *p* = 0.014) on EQ-5D. However, there was only a significant group main effect (F = 26.235; *p* < 0.001) with no effect on time (F = 2.265; *p* = 0.160) and group × time (F = 1.584; *p* = 0.234) interaction on the MFIS scale. Conclusions: The Long-COVID group showed higher lactate concentration compared with the control group during the 12 weeks of the Nordic Walking program. The Long-COVID group presented a decrease in fatigue with respect to the control group according to the MFIS scale, as well as improvement in quality of life after aerobic exercise therapy.

## 1. Introduction

Long-COVID (or Long-COVID-19 syndrome or Long-haul COVID-19) consists of signs and symptoms that develop during or after COVID-19-compatible infection that continues for more than 12 weeks and cannot be explained by an alternative diagnosis [1,2]. Symptoms can often overlap, fluctuate, and change over time; they sometimes come as a result of relapses [3]. Any body system (cardiovascular, respiratory, gastrointestinal, neurological, musculoskeletal, metabolic, renal, dermatological, and haematological) may be affected [4,5]. In addition, psychiatric problems, generalised pain, fatigue, and persistent fever may occur [6,7]. Survivors of COVID-19 infection had a deteriorated physical condition after discharge from hospital [8]. Patients with Long-COVID suffer functional disability similar to patients with severe COVID-19 [9]. Fatigue is the most frequent symptom in Long-COVID syndrome [10]. Patients who were healthcare workers reported fatigue as the most disabling symptom, along with many difficulties in coping with persistent symptoms [11].

In addition, cardiopulmonary deficits and symptoms, such as dyspnoea or fatigue, may persist for months after discharge. However, patients with Long-COVID syndrome report fluctuating aerobic and endurance capacities linked to functional mobility and activities of daily living [12,13]. These patients have shown provable abnormalities in several biomarkers and cardiac, neurological, haematological, renal, hepatic, and endocrine failure [14]. Most patients live with persistent fatigue and reduced exercise capacity not attributable to cardiopulmonary impairment diagnosed by conventional clinical means. Some researchers hypothesise that an impaired systemic oxygen extraction affects people with Long-COVID syndrome [15,16].

To date, the same metabolic deficits have not been found for other coronavirus infections, but very similar impairment assessed with the same outcome measures. Patients with SARS-CoV-1 showed at 1 year that a significant percentage of individuals had reduced 6MWT due to shortness of breath and fatigue. Quality-of-life (QoL) measures (using the SF-36 short form) showed an overall reduction at 3 months, but these had not normalized at 1 year [17].

Middle East respiratory syndrome (MERS) survivors showed a reduction in QoL scores using the SF-36. Similar to SARS-CoV-2, chronic fatigue symptoms were described in 48% of survivors at 1 year [18].

Survivors of non-coronavirus acute respiratory distress syndrome (ARDS) were followed up at 3, 6, and 12 months. Their findings replicated those observed in survivors of both epidemics, with significant weight loss (up to 18% of body weight), reduced 6 min walking test (6MWT), reduced QoL scores, and persistent functional disability 1 year after discharge [19]. The same authors followed this population for 5 years and demonstrated that exercise limitation, physical sequelae, and reduced QoL persisted until that time [20].

In patients with Long-acute sequelae of SARS-CoV-2 infection, significant dysfunction in beta-oxidation and increased blood lactate accumulation during exercise have been found [21]. Also, increased lactate may result from mitochondrial dysfunction in subjects without cardiac or respiratory pathology [22]. This metabolite is always a product of glycolysis, produced in stressful situations like infections. Epigenetic modifications that can lead to metabolic regulation play significant roles in inflammation or cancer [23,24,25]. With major age, the immune system appears to maintain a condition of mild inflammation. The alteration of Angiotensin Converting-Enzyme 2 (ACE2) receptor expression, oxidative stress, adipose tissue- and immune-senescent cell activity, lack of Vitamin D content, as well as a decrease in autophagy and mitophagy may contribute to the high amplitude of the immune response to external challengers in elderly adults [26].

Guntur et al. [21] estimated that exercise intolerance is the most important manifestation of Long-COVID. It is associated with increased arterial blood lactate accumulation and lower rates of fatty acid oxidation during graded voluntary effort exercise tests, suggesting metabolic disturbance and mitochondrial dysfunction. This investigation decided to test for metabolic imbalances in plasma by analysing it from 30 healthy controls and patients who had passed SARS-Cov-2 infection, 16 who had fully recovered, and 29 Long-COVID patients. The Long-COVID patients had plasma metabolites, indicating impaired fatty acid metabolism and dysfunctional mitochondria-dependent lipid catabolism. Plasma metabolites in the Long-COVID syndrome group indicated impaired fatty acid metabolism and dysfunctional mitochondria-dependent lipid catabolism. However, the data were collected retrospectively during the test, presenting gaps to understand this exercise intolerance in Long-COVID patients, and may pave the way for therapeutic intervention [21].

Conventionally, lactate is considered a waste product, which helps maintain temperature and generates the heat produced by muscle movement. It is also known that lactic acid can dissociate into lactate; moreover, a hydrogen ion and CO_2_ can combine with water to dissociate hydrogen ions, which acidifies the environment and causes muscle fatigue [27]. The role of lactate as a regulator of metabolic feedback and as a biomarker molecule was considered. Thus, a significant role of lactate is recognised in many physiological and pathological processes, including the regulation of energy metabolism, immune responses, memory formation, cicatrisation, and tumour development [28]. The fatigue and Long-exertional malaise reported by patients with Long-COVID, was compared to patients with myalgic encephalitis/chronic fatigue syndrome. These subjects had higher lactate levels during exercise testing [29]. Within the recommendations made to the general population for treating some diseases through physical exercise, Nordic Walking is a form of moderate-to-high aerobic exercise that involves the whole body; its beneficial impact is well documented in terms of improved quality of life and development of motor skills [30,31]. Previous reports show positive results in subjects with peripheral arterial occlusive disease after Nordic Walking, significantly improving the distance walked in the 6 Minutes Walking Test (6MWT) [32]. Also, the use of Nordic Walking poles showed a positive result in the reduction in dyspnoea, increased walking distance, and improved quality of life in patients with chronic obstructive pulmonary disease [33]. However, to date, it is unknown whether Long-COVID syndrome modifies aerobic capacity in these patients and the metabolic pathways involved during exercise.

Therefore, the aim of this study was to evaluate and compare lactate concentration in patients with Long-COVID to those who have not developed Long-COVID immediately after the same Nordic Walking sessions designed within a 12-week programme. We hypothesize that due to the metabolic deficiencies produced by Long-COVID syndrome, lactate concentration will be higher in these patients after aerobic exercise therapy intervention.

## 2. Materials and Methods

### 2.1. Study Design

A two-arm non-randomized parallel controlled trial was conducted to determine physiological response to exercise by measuring lactate concentration immediately after each Nordic Walking session in Long-COVID and non-Long-COVID groups.

### 2.2. Patients

Patients in the Long-COVID group were recruited during March 2021, by mass mailing to their AMACOP (Madrid, Spain) e-mail accounts. Finally, the sample was divided into an intervention group (Long-COVID) (*n* = 16) and a healthy control group (non-Long-COVID) (*n* = 13). Patients were confirmed for Long-COVID syndrome by medical staff specially for presenting fatigue or tiredness when performing daily activities and worsening of symptoms after vigorous physical or mental activity. The healthy control group was recruited through advertisements and flyers in the Madrid region. Acceptance of the study and signature of informed consent was required prior to the start of the programme.

The study has been registered at the Registry of Clinical Trials (NCT05453188 on 12/07/2022), and ethical approval was obtained by the Ethics Committee of Hospital Universitario Fundación Alcorcón (Ethical Approval Code 21/175); the study was conducted in accordance with the Declaration of Helsinki of 1964 (last update 2013).

### 2.3. Sample Size

The sample size was calculated using G*Power 3.1.9.7 software for Windows (University of Kiel, Kiel, Germany) [34]. An a priori sample size calculation indicated that 15 Long-COVID patients were needed to obtain statistically significant differences between the intervention and control groups. This a priori sample size was calculated to obtain an effect size of 1.04 of lactate concentrations (statistical power of 80% with type I error set at 5%), based on a previous investigation that obtained these results when they studied the effect of lactate in subjects with Long-COVID syndrome [9]. A target sample size of 40 participants was determined. The characteristics of both groups are shown in Table 1.

Preconditions were established for the selection of the subjects who were to participate in the study: (i) people between 30 and 60 years of age, with a complete COVID-19 vaccination schedule, and (ii) who were willing to undergo initial functional assessment tests and an intervention protocol consisting of physical exercise sessions after which blood samples were taken. In the case of the experimental group, they had to provide a documented diagnosis and have persistent Long-COVID symptoms for at least one year. We recruited Long-COVID syndrome patients and voluntary healthy controls with similar age and anthropometric characteristics, who presented symptomatology at the beginning of the study. Exclusion criteria were: (i) the presence of comorbidities of musculoskeletal nature that contraindicated the practice of the proposed exercises, (ii) the presence of uncontrolled cardiac or respiratory diseases, (iii) the presence of disabling neurological diseases that significantly interfere with the practice of the proposed exercise, (iv) severe anaemia or other comorbidities that significantly interfere with the proposed exercise, (v) diagnosis or symptoms of dysautonomia, (vi) the presence of high levels of fatigue, (vii) desaturations during the practise test that imply a need for exercise oxygen supply, and (viii) ≥3% effort desaturation during exercise.

Patients were asked to submit a recent basic blood test and an electrocardiogram. Participants diagnosed with Long-COVID provided data from previous medical history related to the processes: time of evolution, syndrome characteristics, treatments performed, and recent blood work.

### 2.4. Intervention Protocol

All study participants in both groups underwent a systematic evaluation consisting of anamnesis, a physical examination of the main organs, and systems focused on searching for symptoms interfering with physical exercise. The experimental and healthy groups took part in the Nordic Walking sessions for 90 min (warm-up and teaching the technique, exercise, and cool-down), which were conducted once a week for 12 weeks. At the beginning of each Nordic Walking session, atmospheric humidity and temperature data were recorded. All patients performed a protocol of 45 min of each Nordic Walking session for aerobic response, as previously indicated [35]. After the session, the distance covered by each patient was recorded, and lactate concentration were measured during the first minute immediately after the end of the session. Patients were asked to start Nordic Walking in a staggered manner to facilitate immediate lactate measurement. They started walking, leaving one minute between the start of each participant.

Nordic Walking sessions were completed outdoors weekly from February to May 2023 in Casa de Campo, Madrid, Spain. The instructor started by designing a route without a slope, which was the same during the three months of the intervention.

Instructions were given to all participants during the development of the Nordic Walking sessions (i.e., to complete the time of each session, to walk on flat ground for 45 min, but they could regulate the pace and the distance, which did not have to be the same from one session to the next. They also had to communicate to the health professionals present in every session if they felt dyspnoea, perceived as “they were gasping for breath when they tried to speak”). In addition, all participants were accompanied in all sessions by a nurse specialized in cardiovascular care and two physiotherapists with experience in therapeutic exercise. Patients with Long-COVID should not desaturate during exercise (no more than 3%), and their heart rate values during exercise were taken. Finally, all participants were required to tell the researchers if they felt Long-exertional fatigue immediately after the session or during the following 48 h.

### 2.5. Sample Collection

Lactate samples were collected after multiple 45 min Nordic walking sessions once per week for 12 weeks. A nurse took a blood sample (0.3 microliters) from the participants by capillary puncturing the index finger of the hand in order to obtain the blood lactate concentration data immediately after physical activity (within the first minute) using the Arkrag lactate Pro2 meter.

### 2.6. Outcome Measures

The distance covered by the participants in all Nordic Walking sessions was measured using the POLAR Ignite 2 device (Polar Electro, Kempele, Finland).

In the pre- and Long-Nordic Walking programs, the Modified Fatigue Impact Scale (MFIS), Short Form 36 Health Survey (SF-36), and EURO QoL-5D (EQ-ED) were administered to assess their fatigue and quality of life.

MFIS is a structured, self-report questionnaire modified form of the Fatigue Impact Scale. This instrument provides an assessment of the effects of fatigue in terms of physical, cognitive, and psychosocial functioning. Individual subscale scores can be generated by calculating the sum of specific sets of items. The MFIS consists of 21 items and their administration time is approximately 5–10 min [36].

The SF-36 was used to evaluate health-related quality of life in the sample. It is composed of 35 items, divided into 8 areas: physical function, physical role, emotional role, social function, mental health, general health, bodily pain, and vitality. It also contains an additional item that is not part of any dimension, and which measures the declared evolution of health. The reduced Spanish version was used for this study [37].

EQ-5D is a structured health state descriptive system with five dimensions: mobility, self-care, usual activities, pain/discomfort, and anxiety/depression. These five dimensions together define a total 5^5^ health states formed by different combinations of levels. As a simple instrument, EQ-5D is widely used in various diseases [38].

### 2.7. Statistical Analysis

Continuous data were summarized as mean and standard deviation, and categorical data as counts and percentages. Demographic data were compared using Fisher’s exact test for qualitative outcomes and the Mann–Whitney U test for quantitative outcomes. Between-group differences, a Shapiro–Wilk test was used to compare normally distributed data. A two-way ANOVA (group × session; 2 × 12) was used to compare the effect of lactate and distance covered on groups after the Nordic walk sessions and (group × time; 2 × 2) was used to compare the effect of questionnaires on fatigue and quality of life. The statistical analysis was performed using SPSS 21.0^®^ software (IBM Corp. Released 2012. IBM SPSS Statistics for Windows, Version 21.0. Armonk, NY, USA: IBM Corp).

The significance level was set at *p* < 0.050.

## 3. Results

### 3.1. Subjects’ Recruitment

Thirty-four participants from the AMACOP (*Asociación Covid Persistente in Madrid*), association of Long-COVID in Madrid and the dissemination of advertisements and flyers were interested in participating from February to April 2022. Of these participants, one did not meet the eligibility criteria, and four decided not to participate. Finally, 29 subjects were recruited: 16 from the Long-COVID group, and 13 from the healthy control group.

Baseline characteristics were similar age, height, weight, hospital admission rate, reinfection, and pneumonia as shown in Table 1.

### 3.2. Lactate Concentration

**Figure 1:**
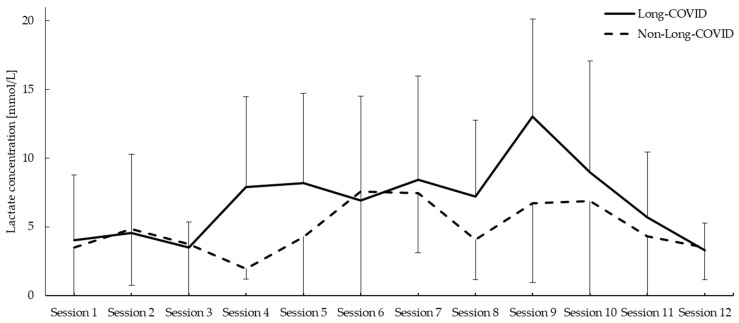
Lactate concentration during the Nordic Walking program.

There was a significant group main effect (F = 5.604; *p* = 0.024) in lactate concentration. However, there was no significant effect of sessions (F = 3.521; *p* = 0.121) with no effect of group × session (F = 1.345; *p* = 0.414) interaction. No differences were found in the sessions (Figure 1).

**Figure 2:**
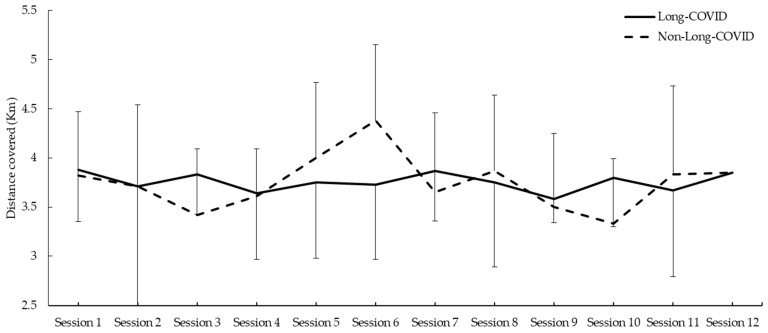
Distance covered during the Nordic Walking program.

Regarding the distances covered during all sessions, no effects were observed between Long-COVID and the healthy control groups (all *p* > 0.050) (Figure 2).

### 3.3. Fatigue

There were a significant group main effect (F = 23.088; *p* < 0.001), time effect (F = 6.625; *p* = 0.026), and group × time (F = 4.632; *p* = 0.002) interaction on SF-36 scale. Also, there were a significant group main effect (F = 38.372; *p* < 0.001), time effect (F = 12.424; *p* = 0.005), and group × time (F = 4.340; *p* = 0.014) interaction on EQ-5D. However, only there was a significant group main effect (F = 26.235; *p* < 0.001) with no effect on time (F = 2.265; *p* = 0.160) and group × time (F = 1.584; *p* = 0.234) interaction on the MFIS scale.

The Long-hoc analysis showed that the Long-COVID group experienced a decrease in fatigue according to the MFIS scale comparing pre- and Long-intervention in the physical subscale [28.62 (±5.31) vs. 23.63 (±7.71); *p* = 0.011], cognitive subscale [25.43 (±9.89) vs. 20.50 (±10.89); *p* = 0.031], and MFIS total score [59.56 (±14.49) vs. 48.62 (±16.81); *p* = 0.009]. Regarding the SF-36 scale, the Long-COVID group recorded an increase in quality of life comparing pre- and Long-intervention in physical functioning subscale [55.00 (±19.23) vs. 64.06 (±19.59); p = 0.001], social functioning subscale [31.56 (±10.28) vs. 51.41 (±14.27); *p* = 0.011], bodily pain subscale [30.78 (±10.05) vs. 50.78 (±12.98); *p* = 0.003], and total SF-36 score [37.22 (±12.61) vs. 48.43 (±14.97); *p* = 0.004]. No differences were shown in other SF-36 subscales (all *p* > 0.050).

These data are related to a decrease in fatigue with respect to the control group, according to the MFIS scale in the different subscales after intervention, and mild improvement in the SF-36 quality of life questionnaire after the intervention, especially in bodily pain, emotional role, and mental health dimensions; the data were recorded according to the EQ-5D health state (Table 2).

## 4. Discussion

This is the first study to address Nordic Walking program as a therapeutic treatment for Long-COVID patients and the first study to measure Long-Nordic Walking lactate concentration. Since the onset of the COVID-19 pandemic, no scientific evidence exists to support the claim that COVID-19 enhances anaerobic metabolism in sports. We refer to energy production without oxygen, mainly used in high-intensity and short-duration activities such as weightlifting or sprinting. COVID-19, on the other hand, is a disease that can negatively affect lung capacity and cardiovascular function, decreasing oxygen supply to the muscles and increasing this deficit during sports.

This trial has shown that, during the 12-week Nordic Walking program, patients with Long-COVID syndrome showed significantly higher levels of lactate concentration immediately after exercise than non-Long COVD subjects, especially in the fourth and ninth sessions (all *p* < 0.005).

According to our findings, the infection with SARS-CoV-2 in Long-COVID syndrome patients may have silenced the oxidative metabolic pathways, producing non-oxidative synthesis pathways and high lactate concentration. Therefore, with little exercise, these patients would considerably increase the anaerobic pathway, producing a large amount of lactate.

It has been adequately proven that blood markers such as high-sensitivity troponin, fibrinogen, blood glucose, C-reactive protein, lactate dehydrogenase, albumin, and ferritin can predict lethal outcomes in patients with COVID-19, and that high lactate dehydrogenase accumulation itself is a potential predictor of disease severity [39,40,41,42].

In our study, patients performed one isolated session of Nordic walking per week. Czer-wińska-Ledwig et al.’s study, using the same methodology, analysed the influence of exercise such as Nordic Walking on lactate dehydrogenase and vitamin D activity in their role in COVID-19 and other diseases, such as muscle damage in multiple myeloma [36]. They concluded that the Nordic Walking program is safe and beneficial for patients with other diseases that cause muscle problems, such as those produced by low lactate dehydrogenase activity that could also affect patients with Long-COVID, indicating muscle damage. These aspects that should be carried out in future studies to extend the knowledge reported in this study. However, it can be noted that our study presented a 12-week Nordic Walking program instead of a 6-week program as presented by Czer-wińska-Ledwig et al. [36], improving the results previously shown.

In our study, the patients conducted one Nordic Walking session per week. Also, a recent study with the same methodology analysed the influence of exercise such as Nordic Walking on lactate dehydrogenase and its role in COVID-19 and other diseases, such as muscle damage in multiple myeloma [43]. In this study, it was concluded that a Nordic Walking program is safe and beneficial for patients with other diseases that cause muscular problems such as those produced by lactate dehydrogenase, which could also affect patients with Long-COVID; these aspects should be carried out in future studies to expand the knowledge reported in this study. However, may be noted that our study presented a 12-week Nordic Walking program instead of a 6-week program as presented by Czerwińska-Ledwig et al. [43], enhancing results previously showed.

Regarding the possible relationship of blood lactate measurement with room temperature, since the present study was conducted in the spring, Smolander et al. [44] suggested that heat (40 °C) did not affect lactate concentration. However, later investigations during proposed exercise suggested that heat stress increased muscle glycogen use and blood lactate level due to increased adrenaline concentrations [45,46,47].

It appears that, after 20 min of exercise in temperatures above 33 °C, increases in sweating and the internal temperature of the athlete are detected. Dehydration and hyperthermia are stressors that stimulate an increase in blood adrenaline concentration and consequently increase glycogenolysis and blood lactate concentration. On the other hand, it may simply be that environmental heat is a stressor to increase glycogenolysis and lactate production independently of dehydration and hyperthermia [47].

Our study did not find a direct relationship between lactate measurement and current temperature; the temperature ranged from 27 °C to 34 °C, and humidity ranged from 20% to 45%. Regarding the number of sessions, our study is consistent in design with that of Reed et al.’s [48] study examining patients with coronary artery disease.

A systematic review on Long-COVID patients found that muscle strength and physical function improved after undergoing an 8-week biweekly physical therapy protocol that included aerobic training, strengthening exercises, and diaphragmatic respiration techniques [49]. Our study, however, involved only 12 sessions instead of 16.

On the other hand, patients infected by the virus who experienced moderate or severe symptoms had more difficulties adapting to exercise; we refer to symptoms such as increased fatigue, muscle weakness, lower exercise endurance, etc. A clinical trial with patients at different stages of severity three months after suffering from COVID-19 showed higher deterioration in those subjects with moderate and severe disease. Of these, 22% showed low exercise capacity [50].

Phenotypes of Long-COVID included mostly cardiovascular or pain symptoms; patients with these symptoms reported lower scores in SF-36 domains of general health, physical functioning, and role limitation due to physical functioning and social functioning [51]. Our results did show a significant difference in these subsections according to SF-36. Other authors found significant positive correlations between lung function parameters with several SF-36 domains. The exercise capacity and health status were considerably lower than that of a normal population 6 months after SARS-CoV-2 infection [52]. This trial demonstrated an improved quality of life according to SF-36 and to the physical function, pain subsections. However, after Nordic Walking program in vitality and mental health, the values of score does not show differences, may be due to the beneficial effect of the program in increasing both physical and mental health. This may be due to the beneficial effect of the program in increasing both physical and mental health. These aspects are consistent with those previously reported by Castro et al. [53] in which they showed that physical exercise intervention with Tai Chi in patients with Long-COVID improved recovery and lung capacity; this work was the first to show that aerobic exercise helps these patients to improve their quality of life. Survivors to COVID-19 had a mental impact, including stress, anxiety and depression according to MFIS scale [54]. This study suggests that the Long-COVID group after the intervention presented a decrease in fatigue with respect to the control group according to the MFIS scale in the different subscales (Table 2). Our results are consistent with another study in which non-invasive brain stimulation paired with a rehabilitation program was effective in reducing fatigue and improving quality of life in people with Long-COVID [55]. Exercise therapy could be a useful tool to understand fatigue and quality of life in these patients who show altered physical characteristics as a result of COVID-19 infection that also influences their daily life.

Several pieces of research used the same outcome measure, using the EQ-5D, obtaining similar results [56,57]. Compared to age- and sex-adjusted reference data, health-related quality of life of persons with Long-COVID was severely impacted [58]. However, non-severe COVID-19 seems to have a negative influence on quality of life, especially in patients with long-term symptoms and with a greater burden from the disease according to EQ-5D [59]. A cohort study demonstrated that respiratory symptoms are frequent at 1 year following COVID-19 and, more importantly, are associated with negative impacts on employment, quality of life, and health care utilization [60].

Given the variety of symptomatology of patients affected by Long-COVID syndrome, there is a tendency to be very cautious when giving recommendations and/or prescriptions for physical exercise. There needs to be more information on the exercise/Long-COVID relationship, and in some cases, the recommendation of such exercise may be beneficial if addressed correctly. Research on biomarkers modified by exercise in Long-COVID could help prevent Long-exertional symptom exacerbation [61,62].

Guntur et al. [21] found that exercise intolerance was the most critical manifestation in Long-COVID patients, and this was associated with increased blood lactate concentration and lower rates of fatty acid oxidation during exercise tests. This suggested metabolic disturbance and mitochondrial dysfunction. The influence of aerobic exercise on the immune response to COVID-19 infection has also been demonstrated in a published case study [63]. Even continuous moderate-intensity home training, resistance training, and combined aerobic and resistance training affected COVID-19 symptoms and severity biomarkers [64]. However, this study was conducted in a randomised design and patients to control possible discomfort during or after exercise. Personalised and supervised physical training is an effective therapy for COVID-19 as it is adapted to the diversity of cases and symptoms. Therefore, supervised, in-person exercise is essential for managing intensity, exercise load, and adherence strategies. We appreciate the patients’ full adherence to this exercise, as Long-effort discomfort is prevalent in Long-COVID patients, and patients reported positive feelings after the exercise [65,66].

In line with the above studies, this trial conforms to the hypothesis of other authors that impaired systemic oxygen saturation affects Long-COVID syndrome patients and that an elevated lactate accumulation immediately after exercise is confirmed [22,67]. The patients in our trial had very good Long-exercise sensations and told us that they were prescribed exercise, but when they tried it, they felt extreme Long-exercise fatigue and needed several days to recover, greatly lowering the level of their pre-exercise activities of daily living. They had very good feelings after Nordic Walking sessions, and adherence to the program during the three months of the trial was unanimous.

Finally, since lactate accumulation produces a decrease in pH, thus worsening physical performance and generating muscular acidosis, it would be advisable to measure this pH in future studies, in addition to other blood biomarkers whose concentrations may be altered in these patients diagnosed with Long-COVID [68]. 

Although there are numerous strengths of the present study—including the study design and the rigorous protocol for data collection—the current investigation has several limitations that should be addressed: (i) The sample size was small because getting patients with Long-COVID to follow exercise programmes is complicated due to the marked Long-exertional fatigue caused by any activity, especially aerobic. For this reason, a larger sample size will be essential to determine the validity of the study and future research is proposed to support this validity. (ii) Also, the non-randomized design of the study could introduce selection bias, although the inclusion criteria were well defined. (iii) The lactate meter indicated some scores Long-exercises outside the metering range when it detected lactate values higher than 25 nmol/L. This occurred only in the Long-COVID group and more frequently on hotter and/or more humid days. However, we could not demonstrate that stressors such as heat and humidity influenced the results in our sample. We repeated the puncture because we could not obtain an objective value when the lactate meter indicated scores outside the metering range. However, we consider that the lactate measurements of the Long-COVID group could have been even higher in the first puncture if the device had recorded higher values. (iv) No other physiological variables, such as heart rate (HR) and partial oxygen saturation (SpO_2_), were measured that could condition such high lactate concentration in Long-COVID subjects. (v) We cannot extrapolate the conclusions to the entire population of patients with COVID or Long-COVID, but only to those with similar clinical and/or functional characteristics of the patients included in the present study. (vi) The gender distribution could impact the generalizability of the study due to the large number of women included in the Long-COVID group. (vii) For future research, we suggest the use of lactate meters that allow, if possible, a more accurate detection of blood lactate. In the Long-COVID group, it was a very frequent occurrence that the device used did not record the first measurement, because it exceeded the maximum; this led to measurements being repeated a second and third time, meaning that possible measurement bias could have been included in the study. vii) These findings indicate that there is an urgent need to preclude persistent or emerging long-term sequelae and provide intervention strategies to reduce the risk of Long-COVID to expand the existing knowledge on neurological, physical, and psychological sequalae, being one of the purposes for future studies in this cohort of subjects with Long-COVID syndrome.

It is essential to remember that COVID-19 is a new disease under constant research. This study is the first to hypothesise and show that Long-COVID disease modifies muscle metabolism due to lung and cardiovascular capacity damage in this patient cohort. It is, therefore, advisable to follow health experts’ guidelines and recommendations and consult a physician or health professional before resuming sports after COVID-19.

## 5. Conclusions

The Nordic Walking program increased blood lactate concentration during aerobic exercise in individuals with Long-COVID syndrome during this moderate-to-high intensity aerobic exercise therapy program. Long-COVID group presented a decrease in fatigue with respect to the control group according to the MFIS scale and mild improvement of quality of life (SF-36 and EQ-5D) after 12 weeks of Nordic Walking exercise therapy program.

From a practical perspective, the outcomes of this investigation suggest that a deeper understanding of the underlying physiological mechanisms in patients with Long-COVID is essential when planning their therapeutic exercise. These results also suggest that anaerobic pathways in Long-COVID patients are the predominant ones, being the first study to show these physiological data, in these patients, during aerobic practice. Exercise therapy could be a useful tool to understand fatigue and quality of life in these patients who show altered physical characteristics as a result of COVID-19 infection that also influence their daily life.

## Figures and Tables

**Table 1:** Baseline characteristics of participants to Long-COVID and non-Long-COVID groups.

		Long-COVID (*n* = 16)	Non-Long-COVID (*n* = 13)	*p*-Value
	Age, mean (SD)	46.13 (7.91)	46.92 (6.00)	0.843
	Height, mean (SD)	65.52 (12.52)	63.14 (13.39)	0.417
	Weight, mean (SD)	166.23 (8.03)	167.25 (7.99)	0.810
Sex	Male, *n* (%)	1 (6.2)	4 (30.8)	0.114
	Female, *n* (%)	15 (93.8)	9 (69.2)	
Hospital admission	No, *n* (%)	15 (93.8)	13 (100.0)	0.424
	Yes, *n* (%)	1 (6.2)	0 (0.0)	
Reinfection	No, *n* (%)	12 (75.0)	13 (100.0)	0.069
	Yes, *n* (%)	4 (25.0)	0 (0.0)	
Pneumonia	No, *n* (%)	13 (81.2)	13 (100.0)	0.125
	Yes, *n* (%)	3 (18.8)	0 (0.0)	

SD; standard deviation.

**Table 2:** Fatigue and quality-of-life characteristics of the participants.

	Pre		Long		Group Main Effect	Time Main Effect	Time × Group Interaction
	Long-COVID	Non-Long-COVID	Long-COVID	Non-Long-COVID	F; *p*-Value	F; *p*-Value	F; *p*-Value
Physical MFIS Score	28.62 (5.31)	7.58 (2.74)	23.63 (7.71)	7.67 (3.09)	28.698; *p* < 0.001	3.484; *p* = 0.089	2.708; *p* = 0.128
Cognitive MFIS Score	25.43 (9.89)	8.75 (3.14)	20.50 (10.89)	8.58 (3.66)	14.818; *p* = 0.003	0.852; *p* = 0.376	0.437; *p* = 0.522
Psychosocial MFIS Score	5.50 (1.89)	1.21 (0.73)	4.50 (1.59)	1.33 (0.68)	43.639; *p* < 0.001	2.200; *p* = 0.166	1.501; *p* = 0.246
TOTAL MFIS Score	59.56 (14.49)	17.58 (6.73)	48.62 (16.81)	17.58 (7.97)	26.235; *p* < 0.001	2.265; *p* = 0.160	1.584; *p* = 0.234
SF-36 Physical functioning	55.00 (19.23)	92.08 (9.64)	64.06 (19.59)	91.66 (15.71)	14.243; *p* = 0.003	8.587; *p* = 0.014	6.947; *p* = 0.023
SF-36 Role physical	16.25 (5.84)	89.58 (19.11)	14.06 (7.58)	81.25 (24.23)	24.484; *p* < 0.001	0.048; *p* = 0.830	1.539; *p* = 0.123
SF-36 Vitality	30.00 (10.32)	59.58 (15.44)	38.43 (17.48)	48.75 (16.25)	13.653; *p* = 0.004	0.032; *p* = 0.861	14.207; *p* = 0.003
SF-36 Role emotional	54.15 (13.65)	88.86 (16.44)	66.65 (17.78)	97.21 (9.63)	9.521; p = 0.010	2.382; *p* = 0.151	0.048; *p* = 0.830
SF-36 Social functioning	31.56 (10.28)	89.58 (15.84)	51.41 (14.27)	89.58 (15.84)	34.015; *p* < 0.001	6.892; *p* = 0.024	4.577; *p* = 0.042
SF-36 Bodily pain	30.78 (10.05)	78.75 (20.68)	50.78 (12.98)	76.04 (15.92)	9.414; *p* = 0.011	4.799; *p* = 0.041	7.676; *p* = 0.018
SF-36 General health	34.06 (14.51)	65.83 (21.58)	39.06 (15.93)	65.40 (13.63)	21.319; *p* < 0.001	0.059; *p* = 0.813	1.483; *p* = 0.249
SF-36 Mental health	56.00 (16.39)	68.33 (8.60)	63.00 (19.67)	74.66 (8.91)	5.275; *p* = 0.042	4.969; *p* = 0.048	0.325; *p* = 0.580
TOTAL SF-36	37.22 (12.61)	75.39 (15.74)	48.43 (14.97)	76.78 (14.73)	23.088; *p* < 0.001	6.625; *p* = 0.026	4.632; *p* = 0.002
TOTAL EURO QoL-5D	41.25 (9.57)	77.50 (10.76)	58.75 (18.64)	81.25 (11.30)	38.372; *p* < 0.001	12.424; *p* = 0.005	4.340; *p* = 0.014

EQ-5D: EURO QoL-5D, MFIS: Modified Fatigue Impact Scale, SD: Standard deviation, SF-36: Short Form 36 Health Survey.

## Data Availability

The data presented in this study are available on request from the corresponding author. The data are not publicly available due to legal restrictions.
